# Extent of Resection, MGMT Promoter Methylation Status and Tumor Location Independently Predict Progression-Free Survival in Adult Sporadic Pilocytic Astrocytoma

**DOI:** 10.3390/cancers11081072

**Published:** 2019-07-29

**Authors:** Christine Jungk, Annekathrin Reinhardt, Rolf Warta, David Capper, Andreas von Deimling, Christel Herold-Mende, Andreas Unterberg

**Affiliations:** 1 Division of Experimental Neurosurgery, Department of Neurosurgery, University Hospital Heidelberg, D-69120 Heidelberg, Germany; 2 Department of Neuropathology, German Cancer Consortium (DKTK), CCU Neuropathology, German Cancer Research Center, Institute of Pathology, University of Heidelberg, D-69120 Heidelberg, Germany; 3 Department of Neuropathology, Charité—Universitätsmedizin Berlin, corporate member of Freie Universität Berlin, Humboldt-Universität zu Berlin, and Berlin Institute of Health, D-10117 Berlin, Germany; 4 German Cancer Consortium (DKTK), Partner Site Berlin, German Cancer Research Center (DKFZ), D-69120 Heidelberg, Germany

**Keywords:** pilocytic astrocytoma, adult, gross total resection, MGMT promoter methylation status, tumor location, progression-free survival

## Abstract

In adults, pilocytic astrocytomas (PA) account for less than 2% of gliomas, resulting in uncertainty regarding the clinical course and optimal treatment, particularly in cases where gross total resection (GTR) could not be achieved. Moreover, information on molecular markers and their prognostic impact is sparse. In order to improve risk stratification, we analyzed our institutional series of 58 patients aged 17 years and older with histology-proven intracranial PA World Health Organization grade I for clinical and molecular prognosticators. Anaplastic and NF1-associated tumors were excluded. O-6-methylguanine-DNA methyltransferase (MGMT) promoter methylation status was determined by pyrosequencing or 450k/850k DNA methylation array. A univariate log-rank test and multivariate StepAIC were applied to identify prognostic factors. The median age was 30 years (range 17–66). Tumors were located in the cerebral/cerebellar hemispheres, midline structures and cerebello-pontine angle in 53%, 38% and 9%. MGMT promoter methylation was present in eight patients (14%). GTR (39/58 patients) significantly reduced the likelihood of tumor recurrence (*p* = 0.0001). Tumor relapse occurred in 16 patients (28%) after a median progression-free survival (PFS) of 135 months (range 6–153 months); there was one tumor-related death. PFS at 5 and 10 years was 67% and 53%. In multivariate analysis, PFS was significantly prolonged in patients with GTR (HR 0.1; CI 0.03–0.37; *p* < 0.001), unmethylated MGMT promoter (HR 0.18; CI 0.05–0.64; *p* = 0.009) and midline tumors (HR 0.21; CI 0.06–0.78; *p* = 0.02). In conclusion, MGMT promoter methylation status and tumor location were identified as novel prognostic factors in adult PAs, pointing at distinct molecular subtypes and detecting patients in need of close observance and intensified treatment.

## 1. Introduction

Pilocytic astrocytoma (PA) World Health Organization (WHO) grade I is the most common pediatric brain tumor, but accounts for less than 2% of adult gliomas [1]. In children, PA usually confers a benign clinical course due to its slow and circumscript growth with 10-year overall survival (OS) of 96% [2,3]. Gross total resection (GTR) is considered to be curative [4] and should be the goal of surgery when feasible, but without inflicting neurological deficits. The majority of pediatric PAs is located in the cerebellum and deep midline structures (brainstem, hypothalamus, opticochiasmatic region). In adults, information on patient outcome and potential prognostic factors is sparse due to the low incidence of PAs in this population (4.8/million/year [2]) and is extracted from a limited number of case reports, case series and one prospective trial including 20 patients (reviewed in [5]). While some series describe an indolent clinical course comparable to pediatric patients [6,7], others report increased recurrence rates and mortality in adults [8,9,10]. In line with the pediatric experience, a recent meta-analysis of seven case series including 254 patients confirmed GTR as a positive prognostic factor in adult PAs, but reported on a mean recurrence rate as high as 31% [5], underlining the need for optimization of risk stratification and treatment. So far, treatment algorithms have been pursued in analogy to those of pediatric patients. However, the optimal treatment, in particular, the timing and modality of treatment after subtotal resection (STR) or biopsy, remains elusive since prognostic and predictive factors apart from GTR are lacking.

In contrast to other glioma subtypes, there are no or, if any, conflicting, data on molecular markers allowing for improved risk stratification, especially in adult PAs [11,12]. In sporadic, i.e., non-neurofibromatosis (NF) type-1-associated cases, PA is a single pathway disease with constitutive activation of the RAS/RAF/MAPK (mitogen-activated protein kinase) pathway [13]. There are two molecular alterations commonly found in pediatric PAs, the BRAF:KIAA1459 gene fusion (“B:K fusion”) and the BRAF V600E mutation [11]. While the B:K fusion has been detected with a high frequency (60–70%) in cerebellar, brainstem and optic pathway cases [14,15], the BRAF V600E mutation is found in a minority of (predominantly extracerebellar) cases (9%) [16] and constitutes a potential therapeutic target [11]. However, their prognostic relevance is still unclear. At the epigenetic level, promoter hypermethylation of distinct genes, amongst others, O-6-methylguanine-DNA methyltransferase (MGMT), has recently been suggested as a potential negative prognostic factor in a series of 18 PAs, including six adult patients [17], but requires further validation.

In search of novel clinical and molecular prognostic factors of this rare adult glioma subtype, we analyzed our institutional series of sporadic, intracranial, adult PAs WHO grade I with particular emphasis on the prognostic significance of MGMT promoter methylation status. The findings of our analysis may help to improve risk stratification and identify patients in need of close observance and intensified treatment.

## 2. Results

### 2.1. Patient Characteristics and Imaging Features

**Figure 1:**
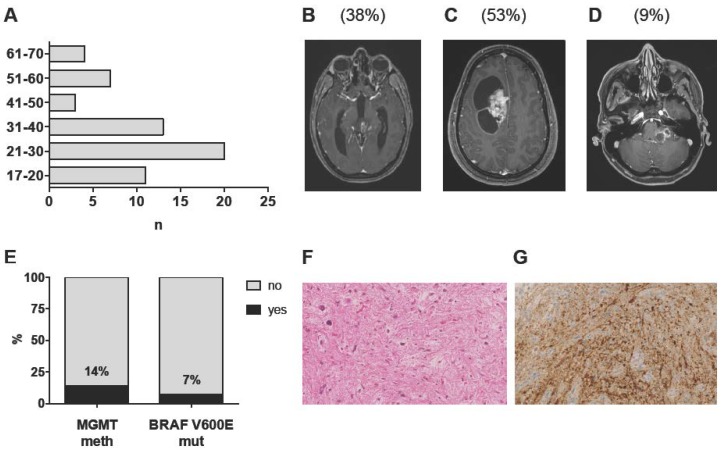
Clinico-pathological characteristics of the complete cohort (n = 58): (**A**) Age distribution at 1st diagnosis in decimal steps. (**B**–**D**) Representative T1 post-contrast MR images of tumors of the midline (**B**), the cerebral/cerebellar hemispheres (**C**) and the cerebello-pontine angle (**D**). Percentages of the respective tumor locations are given in brackets. (**E**) Distribution of MGMT promoter methylation (n = 57 patients) and BRAF V600 mutation status (n = 30 patients). (**F**) Representative hematoxylin/eosin-stained section of a PA WHO grade I (MGMT meth). (**G**) BRAF V600E staining employing a mutation-specific antibody (BRAF V600E positive cells stained in brown). Stainings are displayed in 400-fold magnification.

In total, 76 patients aged 17 years and older with histology-proven intracranial PA diagnosed between 1996 and 2017 were identified from our database. A total of 10 patients were excluded due to insufficient follow-up, one patient due to a history of NF1, three patients due to initial diagnosis of an anaplastic PA and four patients due to discordant histopathological and molecular findings, leaving 58 adult patients with sporadic, intracranial PA WHO grade I for further analysis (Table 1). Median follow-up was 72 months (range 3–259 months). Median age was 30 years (range 17–66 years); only 14 patients (24%) were above the age of 40 (Figure 1A). No gender prevalence was observed (male:female ratio = 0.9). Tumors were equally distributed among the supratentorial and infratentorial compartments (47% vs. 53%) and were located in the cerebral and cerebellar hemispheres, midline structures and the cerebello-pontine angle (CPA) in 53%, 38% and 9% of cases, respectively (Table 1; Figure 1B–D; Table S1). Tumor location was independent of age (supratentorial vs. infratentorial: *p* = 0.83; midline vs. CPA vs. hemispheric: *p* = 0.84) and sex (supratentorial vs. infratentorial: *p* = 0.43; midline vs. CPA vs. hemispheric: *p* = 0.6) (Table 2). Radiographic appearance was heterogeneous with contrast enhancement present in 42 cases (72%) and the typical contrast-enhancing, cystic appearance in 50% of cases only (Figure 1C). The presence of contrast enhancement was independent of age (*p* = 0.93), sex (1.0) and tumor location (supratentorial vs. infratentorial: *p* = 0.31; midline/CPA vs. hemispheric: *p* = 0.17) (Table 2).

### 2.2. Histopathological and Molecular Analysis

Histopathological diagnosis of a PA WHO grade I was based on the presence of the characteristic piloid morphology or a biphasic growth pattern, including areas with loose-textured multipolar cells, eosinophilic granular bodies and/or Rosenthal fibers, low to moderate cellularity and the absence of features of anaplastic transformation (Figure 1F). At 1st recurrence, nine patients (15.5%) underwent re-resection, which confirmed the initial diagnosis in eight patients. In one patient, anaplastic transformation was observed following STR and adjuvant radiotherapy (RT). MGMT promoter methylation status was successfully determined for all but one patient (n = 57). Eight patients (14%) harbored MGMT promoter methylation (Figure 1E). Notably, patients with (MGMT meth) and without (MGMT unmeth) MGMT promoter methylation were comparable with respect to age, sex, tumor location, radiographic appearance and treatment at 1st diagnosis (Table 1). Of note, MGMT promoter methylation was present in the only patient with anaplastic transformation. 

The BRAF V600E mutation was present in two out of 30 patients analyzed (7%; one supratentorial/hemispheric and one infratentorial/midline tumor) (Figure 1E,G). As expected, isocitrate dehydrogenase (IDH) 1 R132H mutation was not detected in any of the 40 patients analyzed (Table 1). 

### 2.3. Clinical Outcome 

At 1st diagnosis, 39/58 (67%) patients underwent radiographically confirmed GTR, 11 (19%) patients STR and eight (14%) patients stereotactic or open biopsy. GTR was more likely in infratentorial than supratentorial tumors (*p* = 0.037; Table 2). Post-operative neurological deterioration persisting >6 months after surgery occurred in 7/58 patients (12%), 6/58 patients (10%) underwent re-operation due to CSF fistula, re-bleeding or disturbed wound healing and 3/58 patients (5%) required permanent CSF shunting due to hydrocephalus (Table S1). In 6/19 (32%) cases with residual disease, adjuvant RT (n = 4), chemotherapy with temozolomide (n = 1) or hyperthermic treatment (n = 1) was administered; otherwise, a wait-and-scan strategy was applied. By chance, the patient treated with temozolomide did not harbor MGMT promoter methylation. Details of treatment at 1st diagnosis are given in Table S1.

**Figure 2:**
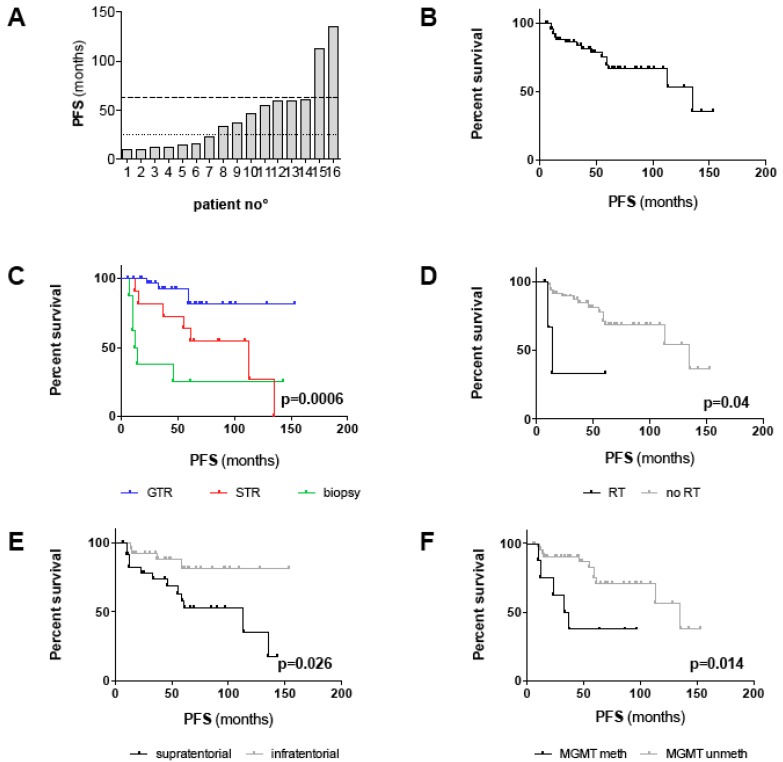

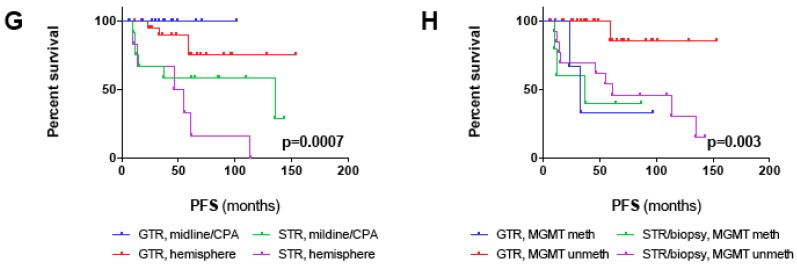
Survival analysis of the outcome cohort including all patients with a follow-up > 24 months and all patients with progression/recurrence irrespective of follow-up (n = 54): (**A**) Diagram depicting time points of tumor progression/recurrence for each individual patient (n = 16). The dotted line marks progression/recurrence within 24 months, the dashed line within 60 months. In 14/16 patients, progression/recurrence occurred within 60 months after 1st diagnosis. (**B**–**H**) Kaplan–Meier plots depicting PFS (in months) for (**B**) the outcome cohort and for (**C**–**F**) all clinico-pathological factors with significant prognostic impact in univariate survival analysis (log-rank test; threshold of significance: 0.05): (**C**) GTR vs. STR vs. biopsy (*p* = 0.0006), (**D**) adjuvant RT vs. no RT (*p* = 0.04), (**E**) supratentorial vs. infratentorial tumor location (*p* = 0.026), (**F**) methylated vs. unmethylated MGMT promoter (*p* = 0.014). (**G**,**H**) Kaplan–Meier plots depicting PFS for patients with midline/CPA and hemispheric tumors (*p* = 0.0007) (**G**) and methylated and unmethylated MGMT promoter (*p* = 0.003) (**H**) stratified for the EOR (GTR vs. STR/biopsy). Note that statistical significances in (**G**) and (**H**) account for comparison of all four groups. P-values for comparison of distinct groups are given in the main text.

Radiographic progression or recurrence was observed in 16/58 (28%) patients within the range of 10–135 months. Interestingly, progression/recurrence occurred rather early in the clinical course, in seven patients within 2 years and in another seven patients within 5 years after surgery (Figure 2A). Nonetheless, two patients with STR conferred an indolent clinical course with progression observed as long as 113 and 135 months after surgery. In 75% of cases, tumor progression was encountered after STR or biopsy, including in three patients with postoperative radio- or chemotherapy; nonetheless, recurrence was also observed in four patients after GTR, with all but one harboring MGMT promoter methylation (Table S1). Thus, the likelihood of tumor progression/recurrence was highly dependent on the extent of resection (EOR; *p* = 0.0001; Table 2); Accordingly, the recurrence rate in completely resected patients was 10%, while the progression rate was as high as 63% in patients with STR/biopsy. Progression/recurrence was also more likely in supratentorial than infratentorial tumors (*p* = 0.019; Table 2) and in patients with MGMT promoter methylation (*p* = 0.032; Table 2). A second recurrence occurred in 6/16 (37.5%) patients, all after initial STR or biopsy. One tumor-related death (1.7%) was reported 75 months after 1st diagnosis.

### 2.4. Prognostic Factors of Progression-free Survival

Since a relatively benign clinical course can be assumed for adult PA patients requiring long-term observation, we set up an “outcome cohort” with a minimum follow-up of 25 months, but also included all 16 patients with radiographic progression/recurrence irrespective of follow-up, for further outcome analysis. Demographic, tumor- and treatment-related characteristics of this cohort (n = 54) resemble those of all patients and are summarized in Table 3. The median follow-up for this cohort was 76.5 months (range 12–259 months). Since only one tumor-related death was noted, the median OS was not reached and thus not considered for survival analysis. Median progression-free survival (PFS) was 135 months (range 6–153 months) and PFS at 5 years and 10 years was 67% and 53% (Figure 2B), respectively. In univariate analysis, supratentorial tumor location (*p* = 0.026), MGMT promoter methylation (*p* = 0.014), STR/biopsy (*p* = 0.0006) and adjuvant RT (*p* = 0.04) were significantly associated with shorter PFS (Table 4; Figure 2C–F). Median PFS for patients with GTR, STR and biopsy were “not reached”, 113 months and 13 months, respectively (Figure 2C). Notably, the unfavorable PFS associated with supratentorial tumor location and adjuvant RT appeared to be the result of a lesser EOR in these patients since both factors were statistically interrelated with the EOR (Table 2). Thus, we performed multivariate analysis applying stepwise forward selection of covariates to adjust for potential confounders. GTR was confirmed as an independent positive prognostic factor of PFS (*p* < 0.001; HR 0.1; 95% CI 0.03–0.37). Moreover, unmethylated MGMT promoter (HR 0.18; CI 0.05–0.64; *p* = 0.009) and midline tumors (HR 0.21; CI 0.06–0.78; *p* = 0.02) were identified as positive prognostic factors, independent of confounding covariates (Table 4). 

Combining the EOR with one of the two other independent prognostic factors helped to improve risk stratification. As depicted in Figure 2G, PFS was non-significantly prolonged in patients with STR/biopsy and midline/CPA tumors compared to patients with STR/biopsy and hemispheric tumors (median PFS 135 months vs. 50 months; *p* = 0.1). Likewise, PFS was non-significantly prolonged in patients with STR/biopsy and MGMT unmeth tumors compared to patients with STR/biopsy and MGMT meth tumors (median PFS 61 months vs. 37 months; *p* = 0.62) (Figure 2H). Noteworthy, MGMT promoter methalytion status seemed to surpass the EOR as a prognostic factor since patients with methylated MGMT promoter conferred poor PFS irrespective of the EOR (median PFS GTR: 33 months vs. STR/biopsy:37 months; *p* = 0.97) (Figure 2H). This is also reflected by the fact that three out of four patients with tumor recurrence after GTR harbored MGMT promoter methylation.

## 3. Discussion

Given the rarity of pilocytic astrocytomas in adults [2], information on the clinical course and potential prognostic factors is sparse. A limited number of studies reported varying results on patient outcome, probably owing to small sample sizes, moderate follow-up, non-standardized postoperative treatment and inclusion of NF1-associated cases and anaplastic histology. Our study was compiled to identify novel clinical and molecular prognostic factors in adult patients with non-NF1-associated, intracranial PA WHO grade I in order to improve risk stratification. We retrospectively analyzed 58 patients, which is, to the best of our knowledge, the second largest institutional series to date [12], and restricted survival analysis to those patients with a minimum follow-up of 25 months and/or radiographic evidence of tumor recurrence/progression (n = 54). 

In this outcome cohort, we observed only one tumor-related death, which translates into a 10-year OS of 96.5%. This compares favorably to other adult PA series [9,12] and is within the range of pediatric series [2,3]. However, progression/recurrence was a frequent event (28%), resulting in moderate 5-year and 10-year PFS of 67% and 53%. This is in line with findings from two of the larger case series, presenting recurrence rates of 30% [8] and 42% [12]. In contrast, a recent case series reported a low recurrence rate of 13% [5] and the only prospective trial observed progression in 1/20 patients (5%) only [7]. Remarkably, in our cohort, 14/16 recurrences (87.5%) occurred within 5 years after 1st diagnosis, but there were also two recurrences 113 and 135 months after STR, questioning recommendations extrapolated from the pediatric experience that magnetic resonance imaging (MRI) surveillance may be omitted after a couple of years, particularly after GTR [4]. Thus, tumor-related mortality was low in our series, but patients experienced a clinically more aggressive course than those reported from pediatric studies and some of the adult studies [6,7]. Of note, 25% of recurrences occurred after radiographically confirmed GTR resulting in a recurrence rate of 10% for completely resected patients, which is considerably increased compared to pediatric patients [4,18]. Nevertheless, GTR was an important prognostic factor for prolonged PFS in our cohort and significantly reduced the likelihood of progression/recurrence, a finding that was also confirmed by multivariate analysis. This is consistent with results from a meta-analysis of seven case series [5] and a recently published institutional series [19], identifying GTR as the only prognostic factor in adult PAs known so far.

Matching the clinical experience, GTR was significantly more common in infratentorial than supratentorial tumors due to enrichment in cerebellar tumors. Likely caused by the interrelation of GTR and infratentorial tumor location, supratentorial location was linked to increased recurrence rates and inferior PFS in univariate survival, which contradicts the indolent course and marginal recurrence rate of adult supratentorial PAs observed in the prospective trial by Brown et al. [7]. In contrast, in the adult PA subgroup analysis of a population-based study from the National Cancer Institute Surveillance, Epidemiology, and End Results (SEER) Program, cerebral/lobar tumors were associated with a higher mortality compared to cerebellar tumors in univariate analysis, although this finding dispersed was not confirmed by multivariate analysis [9]. In order to rule out competing causes, we included tumor location (“supratentorial vs. infratentorial”; “midline vs. CPA vs. hemisphere”) into our multivariate model. Indeed, “supratentorial vs. infratentorial location” shed its prognostic significance. However, midline tumor location (in comparison to hemispheric tumors) was now identified as a prognosticator of prolonged PFS independent of confounding factors such as the EOR. In univariate analysis, midline tumor location did not affect PFS, probably because the simultaneous influence of GTR was stronger than the effect of midline tumor location itself (Figure 2G). Therefore, the independent prognostic significance of midline tumors may be explained by a distinct tumor biology that is responsible for the relatively benign clinical course observed and warrants further molecular investigation.

Unlike other glioma subtypes, there are no molecular prognosticators in adult PAs known to date. We, therefore, searched for robust molecular markers that have already been implemented into routine diagnostic procedures and may help to improve risk stratification in addition to EOR and tumor location. In contrast to diffuse astrocytomas, IDH1 mutation status does not serve as a prognostic factor since PAs have been consistently described as IDH1 wildtype tumors [20]. Accordingly, no IDH1 mutations were detected in our cohort. In pediatric PAs, few molecular alterations, mainly involving the RAS/RAF/MAPK pathway, have been identified with distinct spatial distribution (reviewed in [11]). BRAF is a direct downstream target of RAS, leading to activation of the MAPK pathway. A tandem duplication at 7q34 resulting in a fusion gene between BRAF and KIAA1549 is the most common genetic alteration occurring in 60–70% of pediatric PAs, mainly in tumors of the cerebellum, brainstem and optic pathway [14,15]; however, its reliable detection is technically challenging. Also, a small subset of pediatric PAs (9%) harbors mutations at the BRAF 600 codon with predilection for extracerebellar tumors [16], which can be detected by mutation-specific antibodies [21]. To date, little is known about molecular alterations in adult PAs. In a subset (n = 45) of the largest adult PA series to date, nine cases with B:K fusion (20%) and no cases with BRAF V600E mutations were identified [12]. In our study, we detected BRAF V600E mutations in 2/30 patients (7%) analyzed, a rate that is comparable to the overall low incidence in PAs [16]. Although common in PAs, there is no evidence from the literature that the presence of B:K fusion is associated with outcome, particularly in adult patients as the only study investigating a potential prognostic impact was negative [12]. In pediatric patients, a small number of studies reported conflicting outcome data with respect to B:K fusion status [14,15,22] but analyzed “low-grade gliomas” of different entities (including but not restricted to PAs) together.

In search of novel prognostic factors in adult PAs, we, therefore, focused on MGMT promoter methylation status based on preliminary data derived from a small study of pediatric and adult PAs conducted by Sippl et al. [17]. MGMT promoter methylation was present in 8/18 patients (44.5%) and was associated with a higher rate of recurrence and shorter PFS. In our cohort, MGMT promoter methylation was present in 14% of patients and was independent of age, sex, tumor location, radiographic appearance and EOR. MGMT promoter methylation was significantly correlated with an increased recurrence rate and was identified as an independent prognosticator of shorter PFS in multivariate analysis. It is noteworthy that three out of four patients experiencing tumor recurrence after GTR harbored MGMT promoter methylation. Moreover, the only patient with anaplastic transformation, dying from his tumor after two recurrences, was MGMT promoter methylated. Thus, our findings link MGMT promoter methylation to a more aggressive clinical course in adult PAs and likely to a distinct molecular phenotype even though functional data are lacking to date. In principal, the MGMT gene encodes a DNA-repair protein that removes alkyl groups from the O-6 position of guanine, an important site of potentially deleterious DNA alkylation. Epigenetic silencing of the MGMT gene by promoter methylation reduces DNA-repair activity and enhances the susceptibility of cells to mutagenic events, resulting in inactivation of tumor suppressor genes, genomic instability and tumor formation [23]. This is not specific to gliomas, but accounts for a variety of human cancers [24] and may also account for PAs. Note that, in a recent multicenter series of 102 histologically defined “anaplastic astrocytomas with piloid features” [25], MGMT promoter methylation was observed in 45% of tumors. In high-grade gliomas, epigenetic silencing of MGMT by promoter methylation enhances the sensitivity of tumor cells to alkylating drugs such as temozolomide or nitrosoureas and is, therefore, considered a predictive marker for the clinical response to alkylating chemotherapy [26,27,28]. Whether it may be useful as a predictive marker in adult PAs as well, is beyond the informative value of this study. Neither in our series nor in other adult PA studies, was chemotherapy with alkylating agents part of the standard treatment. Incidentally, the only patient treated with temozolomide after STR in our series did not harbor MGMT promoter methylation.

In this cohort, only five patients received postoperative radio- or chemotherapy. Because of the retrospective nature of this study, it is difficult to deduce the indication for adjuvant treatment in these patients compared to other patients with STR or biopsy alone. Probably, adjuvant treatment was indicated as a result of critical tumor location (e.g., opticochiasmatic system affected in two patients), presenting symptoms and significant residual disease. Extrapolated from the pediatric experience, watchful waiting is routinely performed in adult PAs. In case of residual disease, some authors propose a wait-and-scan strategy with re-intervention in case of tumor progression while others advocate upfront RT although conflicting data on the beneficial or hazardous impact of RT on tumor control and anaplastic transformation exist [7,9,29]. In this regard, additional clinical and molecular prognostic factors can help to identify “high-risk” patients, in particular after STR or biopsy, which may benefit from upfront postoperative treatment. In our series, both clinical (tumor location) and molecular (MGMT promoter methylation status) markers helped to stratify patients with STR/biopsy into favorable and unfavorable outcomes. Thereby, based on our findings, adjuvant treatment may be postponed after STR/biopsy in asymptomatic patients with midline and/or MGMT unmethylated tumors, whereas upfront treatment should be considered in patients with unfavorable prognostic factors. It is of note that this may also apply to patients with methylated MGMT promoter after GTR since median PFS was as poor as for patients after STR/biopsy. Ideally, this should be substantiated by larger, prospective studies; however, this will most likely be hindered by the very low incidence of this disease in the adult population.

Limitations of this study are inherent to its retrospective design with inclusion of patients diagnosed and treated over a time span of two decades. Therefore, timing and modality of adjuvant treatment after STR or biopsy were non-standardized and may have influenced recurrence rates and PFS by unknown confounders, even though only a minority of patients (n = 6) received postoperative treatment. Nevertheless, we tried to minimize a potential bias by applying multivariate survival analysis including known (GTR) and potential (e.g., MGMT promoter methylation status, tumor location, adjuvant RT) confounding factors. Moreover, in order to keep diagnostic uncertainty caused by retrospective patient identification to a minimum, all cases underwent neuropathology review and the EOR was quantified objectively by postoperative MRI rather than by the subjective impression of surgical reports. Given the rarity of adult PAs, we analyzed a considerable number of patients and incorporated, in contrast to all but two series [12,17], molecular data into outcome analysis. Naturally, the optimal treatment and robust algorithms for risk stratification should be determined by prospective trials. However, the very low incidence of adult PAs will necessitate multicenter efforts and the overall indolent course of the long-term follow-up of the disease.

## 4. Materials and Methods 

### 4.1. Clinical Data 

In accordance with local ethics regulations (S-005/2003), our institutional database at the Department of Neurosurgery, Heidelberg University Hospital, Germany, was screened retrospectively for all cases of histology-proven intracranial PA in patients aged 17 years and older with 1st surgery at our department until December 2017. Patients with spinal tumor location, insufficient follow-up, history of NF1 or initial diagnosis of anaplastic PA were excluded from analysis. Medical charts, surgical reports and MRI studies were searched for demographic (age at 1st diagnosis, sex), tumor-related (supra- vs. infratentorial; midline (i.e., brainstem, vermis, hypothalamus, opticochiasmatic region, pineal gland, Foramen Monroi, basal ganglia) vs. cerebello-pontine angle (CPA) vs. hemispheric (both supra- and infratentorial); contrast enhancement) and treatment-related (EOR; adjuvant RT/chemotherapy) factors. EOR was objectively determined on intraoperative, early postoperative or first follow-up MRI and was classified as GTR, STR or biopsy. GTR was defined as no residual tumor nodules on post-contrast T1 (in case of contrast-enhancing tumors) or FLAIR (fluid attenuated inversion recovery; in case of non-enhancing tumors) weighted MRI sequences. Outcome data were retrieved from medical charts, patient contact or registration offices. The follow-up was conducted throughout January 2019.

### 4.2. Histopathological and Molecular Analysis

Histopathological diagnosis of a PA WHO grade I was made based on the WHO classification of Tumors of the Central Nervous System in use at the time of 1st surgery. Additionally, with advances in molecular diagnostics over time, array-based DNA methylation analysis was performed for the more recent cases (23/58 patients; 40%) as described [30]. For all cases (n = 58), MGMT promoter methylation status was analyzed either by pyrosequencing [31,32] (cutoff ≥ 8%) or by 450k/850k DNA methylation array [33]. In the case of a DNA methylation array, the methylation probability cutoff y = 0.358 was used as described by Bady et al. [33] with modifications: for each probe, an individual confidence interval (CI) for MGMT promoter methylation was calculated. If the calculated CI included the cutoff value of 0.358, MGMT promoter methylation status would have been classified as “not determinable”. However, in the present series, MGMT promoter methylation status was determinable for all patients with available 450k/850k data. BRAF V600E and IDH1 R132H mutation status were investigated by immunohistochemistry with mutation-specific antibodies [21,34], but were available for subsets of tumors only.

### 4.3. Statistical Analysis

GraphPad PRISM version 6.0c (Graph Pad Inc., San Diego, CA, USA) was used for statistical analysis of intergroup variance (nonparametric Mann–Whitney test) and contingency (Fisher’s exact test; Chi-square test) of clinico-pathological factors. For survival analysis, an “outcome cohort” including 54 patients with a minimum follow-up of 25 months or radiographic progression/recurrence was used. PFS served as the endpoint and was defined as the time from 1st surgery until radiographic progression/recurrence or last MRI without evidence of tumor progression/recurrence. Since only one tumor-related death occurred, the median OS was not reached and hence not considered as the study endpoint. For the identification of prognostic factors impacting PFS, a univariate log-rank test and multivariate Cox regression analysis were conducted in *R* [www.r-project.org]. For the latter, covariate inclusion was defined by stepwise forward selection conducted by the stepAIC algorithm in the R package “MASS”. Covariates were “age”, “sex”, “supratentorial vs. infratentorial”, “midline vs. CPA vs. hemisphere”, “EOR”, “adjuvant RT”, “MGMT promoter methylation status” and “number of recurrences”. Only cases with all covariates available (n = 53) were included into multivariate analysis. *p*-values < 0.05 were considered statistically significant. 

## 5. Conclusions

Although patients conferred a favorable OS, the recurrence rate was high in this cohort of adult PAs, even after GTR. In addition to the already known prognosticatorGTR, midline tumor location and unmethylated MGMT promoter were identified as novel independent prognostic factors of prolonged PFS that may be considered for individual risk stratification and treatment planning, in particular, in the case of residual disease. Importantly, these novel markers also point to distinct molecular phenotypes that warrant further investigation.

## Figures and Tables

**Table 1:** Patient characteristics of the complete cohort (n = 58); stratified for MGMT promoter methylation status.

	All Patients	MGMT Meth	MGMT Unmeth	*p*-Value
**No. of Patients**	58	8	49	
**Demographic Data**				
Age (years); median (range)	30 (17–66)	41 (20–66)	30 (17–65)	0.23 *
Sex (male:female)	28:30	4:4	23:26	1.0°
**Tumor Characteristics**				
Supratentorial/Infratentorial	27/31	5:3	21:28	0.45°
Midline/Hemisphere/CPA	22/31/5	2:4:2	20:26:3	1.0°
Contrast Enhancement	42	5	36	1.0°
Tumor Cysts	35	5	29	1.0°
**Molecular Data** (available in n patients)				
MGMT Promoter Methylation	8 (57)	8	0	
IDH1 Mutation	0 (40)	0 (6)	0 (33)	
BRAF V600E Mutation	2 (30)	0 (4)	2 (25)	
Secondary anaplastic	1 (58)	1 (8)	0 (0)	
**Treatment at 1st Diagnosis**				
Surgery	58	8	49	
EOR: GTR/STR/Biopsy	39/11/8	3/3/2	35/8/6	0.17 ^&^
Adjuvant Radiotherapy	4	1	3	0.46°
Adjuvant Chemotherapy (TMZ)	1	0	1	1.0°
Others (Hyperthermia)	1	0	1	1.0°
**Treatment at 1st Recurrence**				
Surgery	9	3	6	
EOR: GTR/STR/Biopsy/ND	6/2/0/1	2/0/0/1	4/2/0/0	
Adjuvant Radiotherapy	2	1	1	
Adjuvant Chemotherapy (TMZ)	2	1	1	
**Outcome Data**				
Progression/Recurrence	16	5	11	**0.03°**
2nd Progression/Recurrence	6	2	4	1.0°
Death	1	1	0	0.14°
PFS (months); median (range)	135 (2–153)	35 (10–97)	135 (2–153)	**0.012 ^§^**
Follow-up (months); median (range)	72 (3–259)	72 (38–109)	74 (3–259)	0.27 ^§^

* Mann–Whitney test; ° Fisher’s exact test; ^&^ Chi-square test; ^§^ log-rank test. CPA: cerebello-pontine angle; MGMT: O-6-methylguanine-DNA methyltransferase; meth: methylated; unmeth: unmethylated; IDH1: isocitrate dehydrogenase 1; EOR: extent of resection; GTR: gross total resection; STR: subtotal resection; TMZ: temozolomide; ND: not determinable; PFS: progression-free survival; *p*-values given in bold indicate significance levels below threshold (*p* < 0.05).

**Table 2:** Association studies of potential covariates in the complete cohort (n = 58).

	Age	Sex	supra/infra	ML/Hem/CPA	CE	MGMT meth	EOR	RT	CHT	Recurrence
**Age**	-									
**Sex**	0.62 *	-								
**supra/infra**	0.83 *	0.43°	-							
**ML/Hem/CPA**	0.84 ^&^	0.60 ^$^	**0.043** ^$^	-						
**CE**	0.93 *	1.0°	0.31°	0.17°^,a^	-					
**MGMT meth**	0.23 *	1.0°	0.45°	1.0°^,a^	1.0°	-				
**EOR**	0.99 ^&^	**0.007** ^§^	**0.037** ^§^	0.06 ^§,a^	0.18 ^§^	0.17 ^§^	-			
**RT**	0.79 *	1.0°	0.33°	1.0°^,a^	1.0°	0.46°	<**0.0001** ^§^	-		
**CHT**	NC	1.0°	0.47°	1.0°^,a^	1.0°	1.0°	**0.042** ^§^	1.0°	-	
**Recurrence**	0.4 *	0.24°	**0.019**°	0.56°^,a^	0.67°	**0.032**°	**0.0001** ^§^	0.3°	0.28°	-

* Mann–Whitney test; ^&^ Kruskal–Wallis test; ° Fisher’s exact test; ^§^ Chi-square test; ^a^ for statistical reasons, midline and CPA tumors were analyzed together. Supra: supratentorial; infra: infratentorial; ML: midline; Hem: hemisphere; CPA: cerebello-pontine angle; CE: contrast enhancement; MGMT: O-6-methylguanine-DNA methyltransferase; meth: methylated: EOR: extent of resection (gross total vs. subtotal vs. biopsy); RT: radiotherapy; CHT: chemotherapy; NC: not comparable. *p*-values given in bold indicate significance levels below threshold (*p* < 0.05).

**Table 3:** Patient characteristics of the outcome cohort (n = 54); stratified for MGMT promoter methylation status.

	All Patients	MGMT Meth	MGMT Unmeth	*p*-Value
**Noumber of Patients**	54	8	45	
**Demographic Data**				
Age (years); median (range)	28.5 (17–66)	41 (20–66)	30 (17–65)	0.21 *
Sex (male:female)	27:27	4:4	22:23	1.0°
**Tumor Characteristics**				
Supratentorial/Infratentorial	25/29	5:3	19:26	0.44°
Midline/Hemisphere/CPA	22/28/4	2:4:2	20:23:2	1.0°
Contrast Enhancement	38	5	32	1.0°
Tumor Cysts	32	5	26	1.0°
**Molecular Data** (available in n patients)				
MGMT Promoter Methylation	8 (53)	8	0	
IDH1 Mutation	0 (36)	0 (6)	0 (29)	
BRAF V600E Mutation	1 (26)	0 (4)	1 (21)	
Secondary anaplastic	1 (54)	1 (8)	0 (0)	
**Treatment at 1st Diagnosis**				
Surgery	54	8	45	
EOR: GTR/STR/Biopsy	35/11/8	3/3/2	31/8/6	0.23°
Adjuvant Radiotherapy	3	1	3	0.49°
Adjuvant Chemotherapy (TMZ)	1	0	1	1.0°
Others (Hyperthermia)	1	0	1	1.0°
**Treatment at 1st Recurrence**				
Surgery	9	3	6	
EOR: GTR/STR/Biopsy/ND	6/2/0/1	2/0/0/1	4/2/0/0	
Adjuvant Radiotherapy	2	1	1	
Adjuvant Chemotherapy (TMZ)	2	1	1	
**Outcome Data**				
Progression/Recurrence	16	5	11	**0.045**°
2nd Progression/Recurrence	6	2	4	1.0°
Death	1	1	0	0.15°
PFS (months); median (range)	135 (6–153)	35 (10–97)	135 (6–153)	**0.014** ^§^
Follow-up (months); median (range)	76.5 (12–259)	72 (38–109)	83 (12–259)	0.16 ^§^

* Mann–Whitney test; ° Fisher’s exact test; ^&^ Chi-square test; ^§^ log-rank test. CPA: cerebello-pontine angle; MGMT: O-6-methylguanine-DNA methyltransferase; meth: methylated; unmeth: unmethylated; IDH1: isocitrate dehydrogenase 1; EOR: extent of resection; GTR: gross total resection; STR: subtotal resection; TMZ: temozolomide; ND: not determinable; PFS: progression-free survival; *p*-values given in bold indicate significance levels below threshold (*p* < 0.05).

**Table 4:** Univariate and multivariate survival analysis of the outcome cohort (n = 53).

Covariates of Progression-free Survival	Univariate	Multivariate	
	*p*-Value	*p*-Value	HR (95% CI)
Age; median (high vs. low)	0.41	n.s.	
Sex (male vs. female)	0.39	n.s.	
Supratentorial vs. Infratentorial	**0.026**	n.s.	
Midline vs. Hemisphere vs. CPA	0.85		
- Midline (Ref.) vs. Hemisphere		**0.02**	0.21 (0.06–0.78)
- CPA (Ref.) vs. Hemisphere		**0.038**	0.08 (0.01–0.87)
Contrast-Enhancement	0.3	n.s.	
MGMT Promoter unmethylated	**0.014**	**0.009**	0.18 (0.05–0.64)
GTR vs. STR vs. biopsy	**0.0006**		
- GTR vs. STR	**0.02**		
- GTR vs. biopsy	<**0.0001**		
- STR vs. biopsy	0.25		
- GTR (Ref.) vs. STR/biopsy		<**0.001**	0.1 (0.03–0.37)
Radiotherapy	**0.04**	n.s.	
Chemotherapy	0.11	n.s.	

HR: hazard ratio; CI: confidence interval; n.s.: not significant; Ref.: reference; CPA: cerebello-pontine angle; MGMT: O-6-methylguanine-DNA methyltransferase; GTR: gross total resection; STR: subtotal resection; *p*-values given in bold indicate significance levels below threshold (*p* < 0.05).

## Supplementary Materials

The following are available online at https://www.mdpi.com/2072-6694/11/8/1072/s1. Table S1: Institutional Cohort of Adult Pilocytic Astrocytomas (n = 58): Demographic, Tumor, Molecular and Treatment Characteristics and Patient Outcome.
