# Supplementary material for: Extent of Resection, MGMT Promoter Methylation Status and Tumor Location Independently Predict Progression-Free Survival in Adult Sporadic Pilocytic Astrocytoma
Source: Cancers (Basel). 2019 Jul 29;11(8):1072. doi: 10.3390/cancers11081072 (PMC6721291; doi:10.3390/cancers11081072)
Supplement: Supplementary file 1 [file cancers-11-01072-s001.pdf]

cancers

MDPI

Extent of resection, MGMT promoter methylation status and tumor location independently predict progression-free survival in adult sporadic pilocytic astrocytoma

Christine Jungk, Annekathrin Reinhardt, Rolf Warta, David Capper, Andreas von Deimling, Christel Herold-Mende and Andreas Unterberg

Table 1. Institutional Cohort of Adult Pilocytic Astrocytomas (n = 58): Demographic, Tumor, Molecular and Treatment Characteristics and Patient Outcome.

			Tumor characteristics			Treatment at 1st diagnosis				Outcome			
**No**	**Sex [a]**	**Age (years)**	**Infra-tentorial**	**Location [b]**	**MGMTmeth**	**EOR**	**Surgical Complications[c]**	**Radiotherapy**	**Others**	**No. of Relapses**	**PFS (months)**	**Follow-up (months)**	**Death**
**1**	0	21	1	2	1	STR	Cranial nerve palsy	0	0	2	37	75	1
**2**	0	17	0	3	0	STR	NA	0	0	2	61	259	0
**3**	1	42	0	3	0	Biopsy	0	0	TMZ	2	46	210	0
**4**	0	39	0	1	0	STR	NA	0	0	1	135	211	0
**5**	1	30	1	3	0	GTR	Hemiparesis	0	0	1	59	186	0
**6**	0	61	1	3	0	GTR	0	0	0		153	153	0
**7**	1	20	0	1	0	Biopsy	0	0	0		143	143	0
**8**	0	26	0	3	0	GTR	0	0	0		90	129	0
**9**	1	18	1	3	0	GTR	Dysphagia	0	0		128	128	0
**10**	1	24	1	1	0	GTR	0	0	0		101	130	0
**11**	0	31	0	1	0	Biopsy	0	0	0	2	10	117	0
**12**	1	28	1	3	0	GTR	CSF fistula	0	0		96	114	0
**13**	1	20	1	3	0	GTR	CSF fistula	0	0		26	111	0
**14**	1	51	0	3	1	GTR	0	0	0		97	109	0
**15**	0	18	0	3	0	STR	0	0	0	1	113	119	0
**16**	0	23	1	1	0	STR	Dysphagia, CSF shunt	0	0		109	109	0
**17**	0	36	0	3	0	GTR	0	0	0		44	103	0
**18**	1	24	1	1	0	GTR	0	0	0		18	102	0
**19**	1	18	1	3	0	GTR	Ataxia	0	0		97	97	0
**20**	0	65	1	3	0	GTR	Dysphagia, ataxia	0	0		17	100	0
**21**	0	66	1	1	1	STR	0	0	0		86	87	0
**22**	0	36	1	1	0	STR	Cranial nerve palsy	0	0	1	15	105	0
**23**	0	56	0	3	0	GTR	0	0	0		61	95	0
**24**	1	27	1	3	0	GTR	0	0	0		75	78	0
**25**	1	60	1	2	0	GTR	0	0	0		71	83	0
**26**	0	19	0	0	0	GTR	0	0	0	1	59	83	0
**27**	0	31	1	2	1	STR	Re-bleeding	0	0		64	80	0
**28**	0	24	0	1	0	STR	0	0	Hyperthermia		85	87	0

cancers

**29**	0	20	0	3	1	GTR	0	0	0	1	23	69	0
**30**	1	23	0	3	0	GTR	0	0	0		11	74	0
**31**	0	44	1	1	0	GTR	CSF fistula	0	0		6	68	0
**32**	0	25	0	3	NA	GTR	0	0	0		66	66	0
**33**	1	30	1	1	0	GTR	CSF shunt	0	0		65	65	0
**34**	1	31	0	3	0	GTR	0	0	0		69	70	0
**35**	1	23	1	3	0	GTR	0	0	0		61	61	0
**36**	0	18	1	3	0	Biopsy	0	SRS (13 Gy)	0	2	14	67	0
**37**	0	24	0	1	0	Biopsy	0	FRT (protons, 52,2 GyE)	0		61	61	0
**38**	1	24	0	1	1	Biopsy	0	0	0	1	12	59	0
**39**	1	52	0	3	1	Biopsy	0	FRT (protons, 54 GyE)	0	2	10	52	0
**40**	1	48	1	3	0	GTR	0	0	0		48	48	0
**41**	1	51	0	3	0	STR	0	0	0	1	55	55	0
**42**	0	29	1	3	0	GTR	0	0	0		36	47	0
**43**	1	20	1	1	0	GTR	CSF fistula	0	0		45	45	0
**44**	1	57	0	1	0	Biopsy	0	FRT (photons, 54 Gy)	0		7	42	0
**45**	0	28	1	1	0	GTR	CSF shunt	0	0		49	49	0
**46**	1	32	1	1	0	GTR	0	0	0		44	44	0
**47**	1	64	0	3	1	GTR	0	0	0	1	33	38	0
**48**	1	31	0	1	0	GTR	0	0	0		33	45	0
**49**	1	22	1	2	0	GTR	0	0	0		38	38	0
**50**	0	22	1	1	0	GTR	0	0	0		37	37	0
**51**	0	19	0	3	0	GTR	0	0	0		25	25	0
**52**	0	35	1	1	0	GTR	0	0	0		30	30	0
**53**	1	51	1	1	0	GTR	0	0	0		26	26	0
**54**	0	36	0	3	0	GTR	Wound debridement	0	0		18	18	0
**55**	1	31	1	3	0	GTR	0	0	0		12	13	0
**56**	1	30	0	3	0	GTR	0	0	0		2	3	0
**57**	1	37	1	2	0	GTR	0	0	0		3	3	0
**58**	0	33	0	1	0	STR	0	0	0	1	12	12	0

Sex [a]: (1) female, (0) male. Location [b]: (1) midline, (2) CPA, (3) hemisphere. Surgical Complications: newly acquired neurological deficits persisting > 6 months after surgery. GTR: gross total resection; STR: subtotal resection; CSF: craniospinal fluid; SRS: stereotactic radiosurgery; FRT: fractionated radiotherapy; Gy: Gray; TMZ: temozolomide; PFS: progression-free survival; NA: not available.
